# The impact of basic vs. enhanced Go NAPSACC on child care centers’ healthy eating and physical activity practices: protocol for a type 3 hybrid effectiveness-implementation cluster-randomized trial

**DOI:** 10.1186/s13012-019-0949-4

**Published:** 2019-12-05

**Authors:** Amber E. Vaughn, Christina R. Studts, Byron J. Powell, Alice S. Ammerman, Justin G. Trogdon, Geoffrey M. Curran, Derek Hales, Erik Willis, Dianne S. Ward

**Affiliations:** 1 Center for Health Promotion and Disease Prevention, The University of North Carolina at Chapel Hill, 1700 Martin L. King Jr. Blvd., CB 7426, Chapel Hill, NC 27599-7426 USA; 2 Department of Health, Behavior & Society, College of Public Health, University of Kentucky, 151 Washington Ave, Lexington, KY 40506-0059 USA; 3 Brown School, Washington University, One Brookings Dr., CB 1196, St. Louis, MI 63130 USA; 4 Health Policy and Management, Gillings School of Global Public Health, The University of North Carolina at Chapel Hill, 135 Dauer Drive, CB 7400, Chapel Hill, NC 27599-7400 USA; 5 Department of Nutrition, Gillings School of Global Public Health, The University of North Carolina at Chapel Hill, 135 Dauer Drive, CB 7461, Chapel Hill, NC 27599-7461 USA; 6 Center for Implementation Research, Division of Health Services Research, Psychiatric Research Institute, University of Arkansas for Medical Sciences, 4301 W. Markham Street, Slot # 577, Little Rock, AR 72205 USA

**Keywords:** Children, Obesity prevention, Implementation approach

## Abstract

**Background:**

To prevent childhood obesity and promote healthy development, health authorities recommend that child care programs use the evidence-based practices that foster healthy eating and physical habits in children. Go NAPSACC is an intervention shown to improve use of these recommended practices, but it is known to encounter barriers that limit its impact and widespread use.

**Methods:**

This study will use a type 3 hybrid effectiveness-implementation cluster-randomized trial to compare effectiveness and implementation outcomes achieved from Go NAPSACC delivered with a basic or enhanced implementation approach. Participants will include approximately 25 coaches from Child Care Aware of Kentucky (serving four geographic regions), 97 child care centers with a director and teacher from each and two cross-sectional samples of 485 3–4-year-old children (one recruitment at baseline, another at follow-up). Coaches will be randomly assigned to deliver Go NAPSACC using either the basic or enhanced implementation approach. “Basic Go NAPSACC” represents the traditional way of delivering Go NAPSACC. “Enhanced Go NAPSACC” incorporates preparatory and support activities before and during their Go NAPSACC work, which are guided by the Quality Implementation Framework and the Consolidated Framework for Implementation Research. Data will be collected primarily at baseline and post-intervention, with select measures continuing through 6, 12, and 24 months post-intervention. Guided largely by RE-AIM, outcomes will assess change in centers’ use of evidence-based nutrition and physical activity practices (primary, measured via observation); centers’ adoption, implementation, and maintenance of the Go NAPSACC program (assessed via website use); center directors’, teachers’, and coaches’ perceptions of contextual factors (assessed via self-report surveys); children’s eating and physical activity behaviors at child care (measured via observation and accelerometers); and cost-effectiveness (assessed via logs and expense tracking). The hypotheses anticipate that “Enhanced Go NAPSACC” will have greater effects than “Basic Go NAPSACC.”

**Discussion:**

This study incorporates many lessons gleaned from the growing implementation science field, but also offers opportunities to address the field’s research priorities, including applying a systematic method to tailor implementation strategies, examining the processes and mechanisms through which implementation strategies produce their effects, and conducting an economic evaluation of implementation strategies.

**Trial Registration:**

ClinicalTrials.gov, NCT03938103, Registered April 8, 2019

Contributions to the literature

- This study will combine the Quality Implementation Framework and Consolidated Framework for Implementation Research to create a systematic method for tailoring implementation strategies.
- This study will examine whether these tailored strategies can improve the context and thereby facilitate better implementation.
- This study will examine the cost-effectiveness of two implementation approaches to evaluate whether the added expense of “Enhanced Go NAPSACC” is a worthwhile investment to achieve the desired improvements in centers’ nutrition and physical activity practices.

## Background

Child care is an important setting for childhood obesity prevention because of its reach and influence. In the USA, two-thirds of 3–5-year-olds are enrolled in some form of child care [1]. In countries with universal pre-kindergarten, participation rates are often 95% or higher [2]. Child care can foster healthy eating and physical activity behaviors by serving healthy foods, providing active playtime, limiting screen time, modeling healthy behaviors, and teaching children how to make healthy choices [3]. Leading health authorities, including the World Health Organization and the National Academy of Medicine, have recommendations that call upon child care programs to implement these evidence-based nutrition and physical activity practices [4–6].

However, poor nutrition and physical activity practices are still common. While national health authorities *recommend* use of these practices, they are not *required*. In the USA, few standards are incorporated into state licensing [7, 8]. So, most child care centers serve fried and high-fat foods, excessive juice, and few whole grains; schedules provide inadequate active playtime; staff do not consistently model healthy behaviors; and few teachers provide nutrition or physical activity education [9–12]. It is not surprising that participation in child care has been linked with increased obesity risk [13].

Unfortunately, little is known about how to help child care implement recommended practices [14, 15]. The Nutrition and Physical Activity Self-Assessment for Child Care (NAPSACC) offers a structured process that helps child care programs improve healthy eating and physical activity practices [16, 17]. NAPSACC is implemented with the help of local technical assistants who support child care programs through NAPSACC’s five-step improvement process: self-assessment, action planning, education, technical assistance, and reassessment. NAPSACC’s impact on practices has been confirmed in multiple studies [18–23]. In 2014, NAPSACC was adapted into an online format, reducing the time required of technical assistants from 25 h per center [21] to only 5 h [24]. This online version, known as Go NAPSACC, has been shown to produce similar improvements in practices [24].

While effective, several implementation challenges have been identified, including variation in experience and implementation across NAPSACC Consultants [18, 25]; difficulty converting child care programs into active users [18]; child care programs’ noncompliance with the improvement process [25]; variable director motivation [19]; low staff engagement [23]; turnover in management [19]; insufficient peer learning opportunities to support changes [23]; and lack of funding [22]. The field of implementation science offers several frameworks [26] and strategies [27] that can help systematically identify and address these contextual challenges.

This project will examine whether an enhanced implementation approach could preemptively identify challenges and tailor support to address those challenges, thereby improving Go NAPSACC’s implementation and effectiveness outcomes. Specifically, this study will compare the effects of a basic versus an enhanced approach on child care centers’ implementation of evidence-based nutrition and physical activity practices (primary aim) as well as centers’ implementation of Go NAPSACC, including its adoption, implementation fidelity, and maintenance. Contextual factors will be examined to understand their influence on implementation outcomes under each approach. This study will also examine the effectiveness of these two approaches on changing children’s diet and physical activity behaviors at child care. Finally, cost-effectiveness of these two approaches will be compared. Given that the enhanced approach is designed to identify and address contextual challenges to implementation, it is hypothesized that it will demonstrate better implementation and effectiveness outcomes compared to the basic approach.

## Methods

**Fig. 1:**
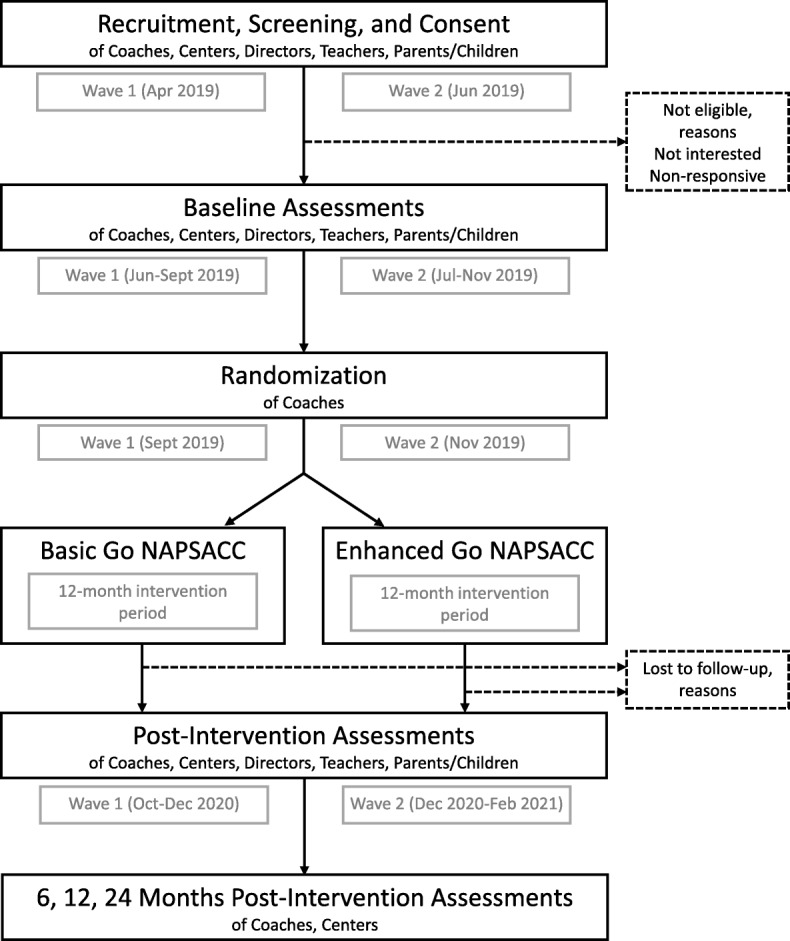
Study timeline

The proposed study will use a type 3 hybrid effectiveness-implementation design with a cluster randomized trial [28] to compare the effectiveness and implementation outcomes of Go NAPSACC delivered with a basic or enhanced approach. The study will be set in four geographic regions of Kentucky (USA): Northern Bluegrass, Southern Bluegrass, Jefferson, and Salt River. These regions’ technical assistance coaches (hereafter referred to as “coaches”) employed by Child Care Aware of Kentucky will assist with center recruitment and then be randomly assigned to deliver Go NAPSACC to their centers using the basic or enhanced approach. Assessment of implementation and effectiveness outcomes will require data collection at baseline, throughout Go NAPSACC implementation, and post-intervention. The study timeline is provided in Fig. 1. Study protocols have been approved by the Institutional Review Board at the University of North Carolina at Chapel Hill and registered at Clinicaltrials.gov (NCT03938103).

### Theories, models, and frameworks

Several implementation science theories and frameworks informed how to deliver Go NAPSACC, how to evaluate its implementation and effectiveness outcomes, and how to identify determinants of its implementation [29]. Development of the enhanced implementation approach was guided by the Quality Implementation Framework (QIF) [30]. This framework has synthesized the implementation literature and offers critical steps for high-quality implementation organized into four phases: (1) *preparing* the organization (e.g., assessing organizational needs, intervention fit, readiness, capacity), (2) *creating a structure* within the organization for implementation, (3) *providing ongoing support* throughout implementation, and (4) *applying lessons learned* to improve future application.

**Fig. 2:**
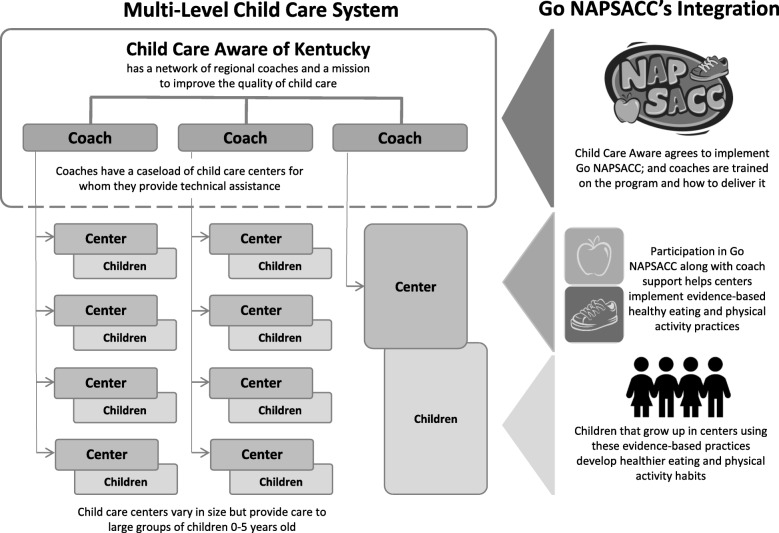
The integration and impact of Go NAPSACC into the multi-level child care system

The RE-AIM framework—specifically the RE-AIM Checklist [31] and updated guidance on the application of RE-AIM [32]—informed the evaluation plan. This framework recognizes that initiatives often work through multiple levels within a system to impact their target. This multi-level approach is consistent with Go NAPSACC’s use of local coaches to deliver the program, which in turn is used by centers to support implementation of best practices (see Fig. 2).

The Consolidated Framework for Implementation Research (CFIR) [33] informed the identification of contextual factors possibly influencing implementation, which were then integrated into the enhanced implementation approach and outcome measures. This framework recognizes that characteristics of the intervention, the outer setting, the inner setting, the individuals involved, and the process of implementation can all impact implementation success. As recommended by CFIR developers, constructs deemed most relevant were identified, focusing on the inner setting of the child care center (e.g., networks and communications, culture, implementation climate, readiness for implementation) and the individuals involved at the center (e.g., knowledge and beliefs about the intervention, self-efficacy).

### Participants and recruitment

Study participants will include coaches, center directors, teachers, and children. They will be recruited in two waves, using a multi-phase process. Child Care Aware of Kentucky, a statewide technical assistance network dedicated to improving the quality of child care (funded by the Child Care Development Block Grant and housed in the Kentucky Cabinet for Health and Family Services), employs two types of coaches: *health and safety coaches* who serve 1–2-star centers, and *quality improvement coaches* who serve 3–5-star centers. In this system, higher star ratings indicate higher quality. Coaches in these four targeted geographic regions will be invited to participate in informational meetings to learn more about the study. Those interested in participating will sign informed consent.

Coaches will share information about the study with centers (randomly selected from their current caseloads) to ensure that centers learn about the study from someone they know and trust. Such strategies are consistent with the “real-world” implementation of Go NAPSACC. Coaches will inform the research team of interested center directors. Research staff will then follow-up by phone with center directors to verify eligibility, review study details, and confirm interest. Eligible centers must have at least one classroom serving 3–4-year-old children, serve lunch, not serve exclusively children with special needs, and have no plans to close in the coming year. Directors must be able to read and speak English.

Research staff will work with center directors to facilitate recruitment within their center. Recruitment of teachers and children will be for measurement purposes only. Go NAPSACC is a center-wide intervention, and as such may affect all classrooms within the center. Directors will identify all 3–4-year-old classrooms. If there are multiple classrooms, one will be randomly selected by research staff for measurement. The lead teacher of this classroom will receive an informational flyer and research staff will follow-up by phone to confirm eligibility, review study details, and confirm interest. To be eligible, teachers must be the lead teacher of the randomly selected classroom and be able to read and speak English. If the teacher is eligible and willing to participate, research staff will collect signed consent from the center director and classroom teacher. They will work with the teacher to distribute informational packets to parents of children in the classroom. Informational packets will describe the study and eligibility criteria and request parental consent for child participation in measures. To be eligible, parents must be able to read and speak English and children must be free of any chronic health condition that severely impacts their diet or physical activity. If needed, the research staff will conduct an onsite visit during normal pick-up times to talk with parents in-person and collect signed consent (as children are too young to consent/assent). Parents of at least three children must consent for the center to remain eligible.

Given the natural turnover in child enrollment, recruitment of children will be repeated 1 year later for post-intervention data collection using similar methods. Thus, the child sample will include two cross-sectional samples.

### Sample size

The sample size calculation for this study focuses on ensuring adequate power to detect change in the primary implementation outcome: centers’ use of nutrition and physical activity best practices. Calculations account for the cluster-randomized design, assuming an average cluster size of six centers per coach and an intraclass correlation of 0.001 (based on previous pilot data). Calculations specify 80% power, an alpha of 0.05, and an effect size of 0.6. The effect size is considered conservative based on published NAPSACC studies showing effects of 0.4 to 1.6 [19, 20, 23, 34]. After accounting for 10% attrition, the final sample size for this study is 97 centers.

### Randomization

Randomization to the basic or enhanced approach will occur once all participants for a wave have been recruited, consented, and scheduled for baseline measures. This timing will minimize the delay between baseline measures and Go NAPSACC implementation. Coaches will serve as the unit of randomization; centers will then follow their coach’s randomization assignment. Coaches will be stratified by geographic region (i.e., Northern Bluegrass, Southern Bluegrass, Jefferson, Salt River) and type (i.e., health and safety vs. quality improvement) prior to randomization to help ensure equal distribution of lower and higher rated centers between study arms. Coaches will then be randomly assigned (1:1) to either the basic or enhanced approach. Coaches, center directors, and teachers will be informed of their study arm assignment. Those directly involved in randomization will be aware of randomization assignments (e.g., statistician who creates the randomization tables, project manager who informs coaches of their assignment, Go NAPSACC specialist who trains coaches on their respective implementation approaches). Investigators, data collectors, and other research staff will remain blinded.

### Go NAPSACC

All coaches will implement Go NAPSACC with their participating centers. Go NAPSACC [24] offers a suite of interactive, online Provider Tools that guide centers through a 5-step improvement process to increase use of healthy eating and physical activity best practices. The self-assessment tool encourages reflection and facilitates comparison of current practices and best practices (step 1). The action planning tool guides goal selection and creation of tailored action plans (step 2). The tips and materials tool offers resources (e.g., videos, educational materials, classroom activities, parent handouts) that support the work in the action plan (step 3). Trainings are available to support knowledge and skill building (step 4). After reaching goals, centers are encouraged to repeat the self-assessment (step 5). While tools can be used independently, coaches are critical to implementation because they orient centers to the online tools, recommend deadlines for various steps, and offer ongoing support. Go NAPSACC provides corresponding Consultant Tools that help coaches monitor their centers’ progress. Table 1 details the basic and enhanced approaches used to deliver Go NAPSACC, described in accordance with TIDieR guidelines [35].

**Table 1:** Implementation models for Basic Go NAPSACC and Enhanced Go NAPSACC with their respective activities/strategies presented in sequence

Basic implementation	Enhanced implementation
Use local technical assistance, specifically Child Care Aware coaches, to assist center directors with implementation of Go NAPSACC.	Use local technical assistance, specifically Child Care Aware coaches, to assist center directors with implementation of Go NAPSACC. QIF Phase 1–Assessment and Adaptation Identification of implementation team • Coaches will meet with center directors (individually) either in-person or by phone to identify potential staff (at least one administrator and two other staff) who can become champions for Go NAPSACC. • Center directors will extend invites; coaches will monitor progress via quick check-ins by phone or email. • Time required is estimated at 1 h. • Intended to enhance Go NAPSACC adoption and implementation by increasing available resources (i.e., staff) to help with implementation, promoting a learning climate where staff input is essential, and expanding networks and communication by having more staff involved in developing the vision and sharing information.
	Readiness Check: • Coaches will meet with each of their implementation teams to introduce the Readiness Check and create a plan for center-wide administration. The Readiness Check will assess the center’s readiness and identify potential barriers and facilitators. The Readiness Check is based off of the CFIR (REF) and assesses characteristics of the inner setting (e.g., communication networks, culture, implementation climate, readiness for implementation) and the staff involved (e.g., knowledge, beliefs, skills, and self-efficacy around child health promotion). • The implementation team will distribute paper copies of the Readiness Check to all center administrators and staff, to be completed anonymously (using either sealed envelopes or a drop box). • Coaches will compile results and present them back to each implementation team in an in-person meeting. Coaches will use results to facilitate a discussion about prioritizing capacity building needs. * Example: Initial results from the Readiness Check may indicate potential challenges related to communication, priority given to child nutrition and physical activity (part of implementation climate), and staff knowledge and skills. After discussing results, the implementation team may decide to prioritize communication as good communication will also be essential for addressing the other challenges.* • Time required to plan and distribute the Readiness Check is estimated at 1 h. Time required to discuss results is estimated at 1–2 h. • Intended to enhance Go NAPSACC adoption and implementation by promoting a learning climate where staff input is valued, acknowledging current limitations in readiness and capacity, and offering tailored supportive resources to address those limitations.
Registration: • Coaches will use their Consultant Tools to send email invites to center directors to register for their Go NAPSACC account. • Time required is 5 min. • Intended to support Go NAPSACC adoption by engaging center directors and providing them access to the Provider Tools.	QIF Phase 2 – Capacity Building and Planning Registration: • Coaches will use their Consultant Tools to send email invites to all members of their implementation teams to register for their Go NAPSACC account. Members from the same implementation team will have linked accounts, allowing all members of the team to see information about their center and its progress on the 5-step improvement process. • Time required to register is about 5 min. • Linked accounts intended to enhance Go NAPSACC implementation by solidifying the available resources (i.e., staff) to help with implementation and facilitating communication between team members.
Orientation: • Coaches will conduct educational outreach visits with center directors in either a one-on-one or small group meetings to introduce Go NAPSACC. • Standardized orientation slides (provided to all coaches) will cover the importance of healthy eating and physical activity in the development of the whole child, Go NAPSACC’s 5-step improvement process and its effectiveness, training on the Provider Tools, and the timeline for the next 12 months (encouraging two cycles through the improvement process). Time will also be provided for hands-on practice with Provider Tools. • Time required is about 1 h. • Intended to support Go NAPSACC adoption and implementation by highlighting the compatibility of Go NAPSACC with other center priorities (e.g., children’s cognitive development and social and emotional health); building awareness of Go NAPSACC’s strength, adaptability, low complexity, and design quality; developing self-efficacy on the use of Provider Tools; and offering a basic plan for implementing Go NAPSACC.	Orientation: • Coaches will conduct educational outreach visits with implementation teams one-on-one during in-person meetings to introduce Go NAPSACC. • Standardized enhanced orientation slides and talking points will be provided to coaches to guide the orientation and ensure that all critical topics are covered. The content will be similar to the orientation used for “Basic Go NAPSACC,” but it will incorporate tailored content based on prioritized capacity building needs. This tailored content will provide guidance on how to build capacity using natural opportunities during Go NAPSACC implementation. * Example: Prioritized capacity building need based on Readiness Check results = Communication. During orientation, the coach will emphasize that good communication allows for a two-way exchange of ideas. The coach will ask the team to identify what channels are currently used for communication with staff and parents and how it could be improved. Finally, the coach will guide the team in planning a communication strategy for announcing the center’s participation in Go NAPSACC, ensuring that it facilitates two-way communication, targets both staff and parents, and makes use of effective communication channels.* • The orientation will also provide time at the end for implementation teams to develop a formal implementation blueprint with key milestones and division of duties over the next 10–12 months of Go NAPSACC implementation. To solidify their formal commitment, team members will sign the final plan. • Time required is 1–1.5 h. • Intended to enhance implementation of Go NAPSACC, use of evidence-based practices, and effectiveness on children’s health behaviors by beginning to address known challenges in the implementation context and thereby increasing readiness and capacity.
Monthly check-ins: • Center directors will use the provided timeline to guide their work through the Go NAPSACC program. • Coaches will check in with center directors monthly either in-person, by phone, or by email to remind directors about Go NAPSACC timelines and to offer facilitation. In-person visits will be strongly encouraged during check-ins that coincide with action planning. Standard agendas and prompts will guide these check-ins and allow the coach to assess progress on the current Go NAPSACC step (assess, plan, take action, learn more, keep it up) and address challenges encountered. • Check-ins will require about 10–30 min each; in-person check-ins may require up to 1 h. • Intended to support implementation of Go NAPSACC, use of evidence-based practices, and effectiveness on children’s health behaviors by prompting the center director about their execution of the program and providing resources (e.g., coach support) in support of changes.	QIF Phase 3 – Launch Go NAPSACC Implementation Monthly check-ins: • Implementation teams will use the plan created during their orientation to guide their work through the Go NAPSACC program. • Coaches will check in with each team monthly (in-person, by phone, or by email) to remind them about Go NAPSACC timelines and to offer facilitation. Similar to “Basic Go NAPSACC,” in-person visits will be strongly encouraged during check-ins that coincide with Go NAPSACC action planning. In addition to the standard agendas and prompts about Go NAPSACC steps (assess, plan, take action, learn more, keep it up), coaches will have access to tailored support guidance that describes how to incorporate advice for prioritized capacity building needs throughout the improvement process. * Example: Prioritized capacity building need = Communication. During the assessment check-in, the coach will prompt the team to share results of the initial self-assessment and elicit feedback from staff and parents about potential goals. During the planning check-in, the coach will inquire about staff and parent feedback received and advises on how to incorporate that into goal selection and action planning. The coach will also highlight critical steps in the action plan where there are natural opportunities to promote communication and how to find helpful resources in the tips and materials library to use for that communication. During take action check-ins, the coach will follow-up about these critical steps, offer guidance about any communication challenges encountered, and reminds the team about upcoming communication opportunities. During the keep it up check-in, the coach will encourage the team to reflect on the communication strategies used, how they impacted the effectiveness of their communications, and how the same strategies could be applied to help with communication about other issues (outside of Go NAPSACC).* • Tailored check-ins are estimated to require 20–30 min each; in-person check-ins may require up to 1 h. • Intended to enhance implementation of Go NAPSACC, use of evidence-based practices, and effectiveness on children’s health behaviors by addressing known challenges in the implementation context and thereby increasing readiness and capacity.
	QIF Phase 4 – Apply Lessons Learned Cross-center meetings: • Coaches will facilitate cross-center implementation team meetings every 3 to 4 months. Coaches will have the option of hosting either in-person meetings or video conference calls. These meetings will bring together the implementation teams from different centers and use standard discussion guides to encourage reflection on their efforts, sharing of lessons learned, and peer support and encouragement. • These meetings will require about 1 h each. • Intended to enhance use of evidence-based practices as well as effectiveness on children’s health behaviors by building self-efficacy of implementation team members.

### Basic implementation

“Basic Go NAPSACC” represents the traditional implementation approach. Coaches will use their Consultant Tools to invite center directors to register for a Go NAPSACC account. Then, coaches will provide an in-person Go NAPSACC orientation to center directors using standardized slides that cover the importance of healthy eating and physical activity in the development of the whole child; Go NAPSACC’ 5-step improvement process and its effectiveness; training and hands-on practice with the Provider Tools; and a 12-month timeline for implementation. Afterward, coaches will check-in monthly with center directors (in-person, by phone, or by email) about progress and challenges using standard agendas and prompts.

To prepare for Basic Go NAPSACC implementation, coaches will complete a 3-part training delivered over 2 days by a Go NAPSACC specialist (a masters-trained nutrition educator with 2 years of experience facilitating the implementation of Go NAPSACC in multiple states). The first part of the training will introduce coaches to Go NAPSACC, best practices, the 5-step improvement process, and Provider Tools. It will be conducted in-person and last 1.5 h. As a homework assignment, coaches will create a fictional child care provider account and practice using the Provider Tools. This assignment will take approximately 30 min. Coaches will return the following day to learn about Consultant Tools, the basic implementation approach, and how the Consultant Tools will help them manage their caseload of centers. This training session will also be conducted in-person and last 1–1.5 h.

### Enhanced implementation

Coaches randomly assigned to “Enhanced Go NAPSACC” will deliver Go NAPSACC using a model guided by the QIF’s four-phase implementation approach [30] and the CFIR [33].

In phase 1 (*preparing*), coaches will help each center director identify an implementation team with at least one administrator and two staff. Coaches will meet with each team briefly to introduce the Readiness Check (a paper-based readiness and capacity assessment based on CFIR [33]) and create a plan for its center-wide administration. Coaches will summarize data from Readiness Check surveys and present it back to the team at a subsequent in-person meeting to guide a discussion of priority capacity building needs. Phase 1 activities will take about 2 months to complete.

In phase 2 (*creating a structure*), coaches will use their Consultant Tools to invite members of these teams to register for a Go NAPSACC account. The Go NAPSACC system allows multiple people from one center to create linked accounts. Once registered, coaches will provide an in-person orientation to each team using standardized slides. Slides will be similar to those used in the basic approach but will offer tailored content that addresses possible capacity building needs. Time will also be provided for the team to develop a 10-month workplan for completing two cycles of Go NAPSACC’s improvement process.

In phase 3 (*providing ongoing support*), coaches will check-in with teams monthly in-person, by phone, or by email to inquire about their progress and troubleshoot challenges. Coaches will incorporate tailored support to continue capacity building efforts initiated during orientation. In addition to the standard check-in agendas and prompts, coaches delivering Enhanced Go NAPSACC will have access to tailored support guidance for each capacity building need.

In phase 4 (*applying lessons learned*), coaches will organize cross-center team meetings every 3 to 4 months to bring together teams from the same region to reflect on their efforts, share lessons learned, and offer support to one another. Meetings will be conducted in-person or by video conference using standard discussion guides.

To prepare for Enhanced Go NAPSACC implementation, coaches will participate in a 5-part training delivered over 3 weeks. The training will be delivered by the same Go NAPSACC specialist that delivers the training for Basic Go NAPSACC. The first part of the training will be identical to that used for Basic Go NAPSACC, also being conducted in-person and lasting 1–1.5 h. Like Basic Go NAPSACC, coaches will also complete the 30-min homework assignment to practice using Provider Tools. Coaches will return the following day for the third part of the training, which will introduce Consultant Tools and then guide them through the enhanced implementation approach and possible capacity building needs. This training session will be conducted in-person and last 3.0 h. About 1 week after these trainings, coaches will participate in a 1–1.5-h training on the Readiness Check, including content, administration, scoring, and presenting results back to implementation teams in centers. About 1 week later, coaches will participate in another 1–1.5-h training focused on providing tailored support, including capacity building content and resources available to support that work. Both trainings will be conducted via webinar to facilitate questions and personal interaction with coaches. Ongoing support will be provided via monthly group video conference calls with the Go NAPSACC specialist, each lasting about 1 h. Coaches will also receive 3 one-on-one coaching sessions (one every 3–4 months) with the Go NAPSACC specialist, conducted by phone and lasting about 1 h.

### Outcome measures

Outcome measures will be collected throughout the study, starting with baseline measures, continuing through Go NAPSACC implementation, and concluding with post-intervention measures. Measures will be multi-level and include assessment of coaches, centers, directors, teachers, and children. The primary outcome will be change in centers’ use of healthy eating and physical activity best practices from baseline to post-intervention. Additional measures will be used to assess centers’ implementation of Go NAPSACC as well as implementation context at baseline and post-intervention. Effectiveness of Go NAPSACC in changing children’s diet and physical activity behaviors at child care will be assessed using child-level measures. Finally, costs of delivering Basic and Enhanced Go NAPSACC will be captured to evaluate cost-effectiveness. Data will be collected using a combination of observation and physical measures (collected during a 1-day visit to each center), extraction of website data through standard reports, tracking forms, and self-administered surveys.

#### Use of best practices

Centers’ use of healthy eating and physical activity best practices will be assessed with the Environment and Policy Assessment and Observation (EPAO) [36], which uses direct observation and document review to capture child care practices (e.g., foods and beverages provided, feeding practices, feeding environment, menus, time provided for active play and outdoor play, indoor and outdoor play environment, teacher active play practices, screen availability, teacher screen practices, education and professional development, and policy). This measure has good inter-rater reliability [36] and sensitivity to change following interventions [18, 37, 38]. EPAO data will be collected during the 1-day visit using the center’s randomly selected classroom. The classroom will be observed for a full day (from 7–8 a.m. to 5–6 p.m.), except during naptime when research staff will conduct the document review. The EPAO scoring rubric will be used to calculate one overall nutrition and physical activity environment score (score range = 0–60, higher scores indicate greater use of best practices).

#### Implementation of Go NAPSACC

RE-AIM dimensions that focus on setting-level implementation outcomes have been prioritized, including adoption, implementation fidelity, and maintenance. As recognized by RE-AIM, these dimensions can apply to multiple levels, which in this study include centers (the organizations participating in the program) and coaches (the intervention agents delivering the program).

*Adoption* is defined as the absolute number, proportion, and representativeness of organizations and intervention agents that agree to participate and initiate the program [32]. Coaches’ recruitment tracking forms will capture centers approached, methods used to contact, reasons for not participating (i.e., not eligible, not interested, unable to establish contact), and referrals. Screening forms, completed by research staff, will capture center eligibility and interest, selection of a 3–4-year-old classroom, engagement of a teacher, and distribution and collection of parent consent. Go NAPSACC’s Registration Report will capture all centers that register for a Go NAPSACC account—indicator of program initiation. Center demographic data captured in this report will be compared to similar state-maintained data on all licensed child care programs to evaluate the representativeness of adopters to other centers in Kentucky. Similar recruitment tracking information and Go NAPSACC website data will be captured for coaches.

*Implementation fidelity* is defined as the extent to which the organization participates in the program and the intervention agent delivers the program as intended [32]. Go NAPSACC’s Detailed Activity Report will capture centers’ participation in Go NAPSACC’s 5-step improvement process, specifically completion of self-assessments, selection of goals, and creation and completion of action plans. Go NAPSACC’s TA Activity Report will capture coaches’ delivery of Go NAPSACC, including the contacts for each center, dates and length of those contacts, support for specific steps in the improvement process, and health content (e.g., healthy eating, physical activity). These reports will capture the fidelity of centers’ participation in Go NAPSACC and coaches’ delivery of basic and enhanced approaches.

*Maintenance* is defined as the extent to which behavior change is sustained 6 months or longer following intervention, as well as the extent to which a program becomes institutionalized in routine practices [32]. Continued use of Go NAPSACC and the long-term changes achieved will be monitored using Go NAPSACC’s Detailed Activity Reports and TA Activity Reports, assessed at 18, 24, and 30 months after initiation of Go NAPSACC (i.e., 6, 12, and 24 additional months post-intervention) [39]. Completion of additional self-assessments will indicate that centers are still using Go NAPSACC. Later self-assessments can be compared to earlier ones to evaluate whether changes are maintained. Logging of additional TA activities will indicate that coaches are continuing to deliver Go NAPSACC.

#### Contextual factors influencing implementation

As recommended by the CFIR framework [33], the most salient constructs were identified based on barriers identified in previous NAPSACC studies [18, 19, 22, 23, 25] and our extensive and ongoing work implementing Go NAPSACC. Prioritized constructs were operationalized for the child care setting and a nutrition and physical activity intervention. Self-administered surveys completed by directors, teachers, and coaches at baseline and post-intervention will be used to assess these constructs. Survey items draw from existing scales, including Fernandez’s Inner Setting Survey (ISS) [40], the Organizational Readiness for Change (ORC) survey [41, 42], and Seward’s Theoretical Domains Framework Questionnaire (TDFQ) for child care [43–45]. The ISS and ORC use a 5-point Likert scale, while the TDFQ uses a 7-point Likert scale (where 1 = strongly disagree and 5 or 7 = strongly agree). Table 2 identifies prioritized constructs, source measures and subscales, and data source (i.e., directors, teachers, and/or coaches).

**Table 2:** Measurement of implementation context

CFIR construct	Source	Asked to:	
		Directors and teachers	Coaches
Networks and communications	ORC: Organizational Climate–Communication [41, 42]	Yes	No
Culture	ISS: Culture, Culture Stress, Culture Effort [40]	Yes	No
Implementation climate	ISS: Implementation Climate [40]	Yes	Yes
Readiness for implementation			
Leadership engagement	ISS: Leadership Engagement [40]	Yes	No
Available resources	ISS: Available resources [40]	Yes	Yes
Access to information and knowledge	ORC: Resources–Training [41, 42]	Yes	Yes
Knowledge and beliefs about intervention	TDFQ: Knowledge [43–45]	Yes	Yes
	TDFQ: Beliefs and Consequences [43–45]	Yes	Yes
Self-efficacy	TDFQ: Beliefs about Capabilities [43–45]	Yes	Yes

#### Children’s diet and physical activity

Children’s dietary intakes at child care will be captured using the Diet Observation at Child Care protocol [46]. This protocol relies on certified data collectors to estimate and record the amount of food and beverages served, wasted, exchanged, and remaining for each child for each meal and snack eaten at child care. Data will be collected during the 1-day visit. Data collectors will randomly select three of the participating children to observe (maximum allowed per protocol). Data will be entered into the Nutrition Data System for Research (NDSR, University of Minnesota) to estimate intakes of energy, macro- and micronutrients, and servings of different food groups. Then, the Healthy Eating Index 2015 [47] scoring algorithm will be applied, which rates diet quality on a scale of 0–100, where higher scores indicate greater compliance with national dietary guidelines.

ActiGraph GT3X+ accelerometers (ActiGraph, Pensacola, FL) will be used to estimate children’s physical activity at child care. Data collectors will place accelerometers on up to five participating children at the beginning of the 1-day visit. Monitors will be removed when children leave. Data will be downloaded and processed to assess wear and physical activity outcomes. Age-appropriate cut-points will be applied to calculate minutes per hour of moderate to vigorous physical activity, active play, and sedentary time [48–50].

#### Cost-effectiveness

Cost of implementing Go NAPSACC using the basic and enhanced approaches will be tracked from the perspective of Child Care Aware of Kentucky, the organization that employs the coaches. Coaches will keep records of time spent implementing Go NAPSACC, including both planning time and all direct contacts using the Go NAPSACC website’s Add TA Activity, making sure to also note any supplemental expenses.

#### Participant characteristics

Participants will complete brief demographic surveys to assess age, sex, race, ethnicity, education, and income. For center directors, supplemental questions will be asked about center characteristics (e.g., years of operation, quality rating, participation in subsidy programs). For child participants, the survey will be completed by parents and capture date of birth, which will be used to calculate exact age on the day of measurement. Also, children’s height and weight will be measured during the 1-day visit. Measures will be taken while children are in light clothing with shoes removed. Height will be measured to the nearest 1/8 inch using a Seca stadiometer (Seca Corporation, Columbia, MD); weight will be measured to the nearest 0.1 pound using a Tanita 800BWB scale (Tanita Corporation, Tokyo, Japan). Height and weight will be used to calculate BMI percentile and z-score using the SAS code provided by Centers for Disease Control and Prevention [51].

### Statistical analysis

The primary analyses will compare changes in centers’ use of nutrition and physical activity best practices, baseline to post-intervention, between centers receiving Basic Go NAPSACC and those receiving Enhanced Go NAPSACC. Analyses will use Generalized Linear Mixed Models (GLMM) that account for clustering of centers under coaches. The GLMM will include a random intercept for coach, fixed effects for the baseline value of the primary outcome and the intervention, and covariates relevant to change in EPAO scores (identified a priori). Analyses will also explore interaction between treatment group and other covariates, and change in completers only. Baseline demographics and EPAO scores will be compared between completers and non-completers to inspect for potential bias. In addition, data will be assessed to evaluate whether data are missing completely at random, missing at random, or missing not at random. When appropriate, multiple imputations [52] will be employed to assess the sensitivity of results [53].

The analyses of adoption, implementation fidelity, and maintenance, described above, will use primarily descriptive statistics.

A multilevel structural equation model approach, as described by Preacher and Thomas [54], will be used to explore how contextual factors influence implementation. Such models are uniquely suited to account for clustering of data within centers that violate the assumption of independence of observations [54–58]. The model will use a two-level framework with center- and coach-level variables. Baseline contextual factors (Table 2) that predict changes in centers’ use of nutrition and physical activity best practices will be examined first. Mediation analysis will be employed to determine whether changes in centers’ use of nutrition and physical activity best practices (an a priori condition for mediation) are explained by changes in contextual factors.

The analyses of child-level effectiveness outcomes (i.e., diet, physical activity, BMI) will be conducted with an intent-to-treat approach using repeated measures linear mixed effects models [59, 60] to account for the use of two cross-sectional samples of children, each nested within a center which is nested under a coach. The fixed effects within these models will include categorical time (baseline, post-intervention), trial arm, and their interactions. Distinct correlated random center effects for each time period will be fit to ensure an appropriately modeled covariance structure for the outcomes and thus valid inference. This will allow for the possibility of a separate intraclass correlation at each time point, as well as different correlations among outcomes from subjects in the same center but at different points in time. Tests will compare mean changes from pre- to post-intervention between intervention and control accounting for clustering and covariates.

The cost-effectiveness analyses will be used to evaluate whether Enhanced Go NAPSACC is cost-effective compared to Basic Go NAPSACC. Time estimates, extracted from TA Activity Reports, will be combined with coaches’ salaries to calculate staffing costs. Supplemental expenses, such as printing and mileage, will be added to determine the total cost of implementing Basic Go NAPSACC and Enhanced Go NAPSACC. The incremental cost of delivering Enhanced Go NAPSACC will be divided by the incremental change in effectiveness measured by the unit increase in EPAO scores (relative to Basic Go NAPSACC) to quantify the incremental cost-effectiveness ratio.

### Data monitoring

All phases of this study will be monitored by a data safety officer, an independent consultant who has worked with investigators to develop a comprehensive plan for monitoring recruitment, data collection, implementation of Go NAPSACC, and data analysis. During recruitment and baseline data collection, the data safety officer will receive monthly updates on subject accrual and a formal report at the end of each wave detailing final enrollment and baseline measurement. Once implementation begins, the data safety officer will receive quarterly reports about adoption, implementation fidelity, adverse events, and retention of centers for post-intervention measurement (when applicable). Given the study’s minimal risks, failure to recruit participants is the only reason for stopping the study early.

Data will be collected and stored in a manner that protects participant confidentiality. Participants will be assigned an ID number that will be used on all paper surveys and electronic records with participant data. Identifying information collected during the study will be stored separately on secure and password protected servers. Results of the study will be summarized and shared with the research community as well as with community partners. A final study dataset will be made available but will require a data sharing agreement with the principal investigator (DW) and the University of North Carolina Chapel Hill.

## Discussion

The field of implementation science offers many lessons that need to be incorporated into child care–based intervention studies, as most child care–based research to date has focused primarily on efficacy, and to a lesser extent, effectiveness [14, 61]. True child care–based implementation studies have only recently emerged, primarily in Australia [62–64]. Hence, existing child care studies offer limited information about implementation outcomes (e.g., adoption, implementation fidelity) and they lack systematic assessment of context (e.g., culture/value for health, relative priority of nutrition and physical activity, leadership buy-in, available resources, knowledge and beliefs of staff). This study will not only examine context but consider it from multiple perspectives, including the centers (i.e., center director and teacher perceptions) and the community technical assistance agencies (i.e., coach perceptions).

While this study will add greatly to the child care field, it will also contribute to important gaps in the field of implementation science. Specifically, this study will apply a systematic method to tailor implementation strategies, examine the mechanisms through which implementation strategies produce their effects, and conduct an economic evaluation of implementation strategies [65].

This study will compare the effects of two implementation approaches, Basic and Enhanced Go NAPSACC, both of which use multifaceted strategies to support centers’ participation in Go NAPSACC. While the traditional approach in Basic Go NAPSACC it has been effective, several contextual barriers to widespread use have also been noted [18, 19, 22, 23, 25]. The integration of QIF [30] and CFIR [33] into the enhanced approach offers a systematic method for identifying contextual barriers and then tailoring key implementation strategies. The integration of QIF and CFIR in the enhanced approach offers the opportunity to evaluate whether these frameworks can offer an effective and systematic method for tailoring intervention strategies, using the child care setting as a test case.

Additionally, this study offers the opportunity to evaluate the mechanisms through which the implementation approaches have an effect [66]. As noted by Williams, there is a lack of multi-level mediational analyses examining how strategies influence implementation outcomes [67]. This study will collect detailed data in TA Activity Logs about coaches’ implementation efforts, including the number of contacts, method of contact (e.g., phone, email, in-person), and content. It will also assess implementation context at baseline and post-intervention for coaches and centers. These data, together with data on centers’ use of evidence-based practices, will allow mediational analyses of whether Enhanced Go NAPSACC was more effective in addressing contextual barriers—thereby enabling centers to improve their practices—compared to Basic Go NAPSACC. It also allows examination of how implementation context from the coaches’ perspective influences their implementation of Basic and Enhanced Go NAPSACC as well as its ultimate impact on centers’ use of evidence-based practices.

This study will also provide a careful economic evaluation of Basic and Enhanced Go NAPSACC. Harvard University’s CHOICES project has examined the costs of implementing the original in-person and paper-based version of NAPSACC, with costs varying widely between states (ranging from $36–$101 per child) [68–72]. While use of the online version, Go NAPSACC, is growing, costs and potential savings have not been evaluated. This study will help confirm whether the translation of the program into an online format helps reduce costs of implementation, as suggested from the initial Go NAPSACC pilot [24]. It will also capture the additional costs associated with Enhanced Go NAPSACC and evaluate whether the added costs are worthy of the investment. Such information is critical for states considering whether to implement Go NAPSACC, but also informs researchers trying to make pragmatic decisions when planning implementation approaches [73–75].

At the time of submission, participants in wave 1 have completed baseline data collection, while participants in wave 2 are just beginning baseline data collection. After baseline data collection on both waves is complete, data cleaning will begin. There is strong enthusiasm and support for Go NAPSACC, regardless of implementation approach, from Child Care Aware of Kentucky and hopes to train coaches statewide to disseminate Go NAPSACC.

## Data Availability

Not applicable.

## Abbreviations

CFIR: Consolidated Framework for Implementation Research
EPAO: Environment and Policy Assessment and Observation
GLMM: Generalized Linear Mixed Models
ISS: Inner Setting Survey
NAPSACC: Nutrition and Physical Activity Self-Assessment for Child Care
ORC: Organizational Readiness for Change
QIF: Quality Implementation Framework
TDFQ: Theoretical Domains Framework Questionnaire
